# Effects of forcefield and sampling method in all-atom simulations of inherently disordered proteins: Application to conformational preferences of human amylin

**DOI:** 10.1371/journal.pone.0186219

**Published:** 2017-10-12

**Authors:** Enxi Peng, Nevena Todorova, Irene Yarovsky

**Affiliations:** School of Engineering, RMIT University, Melbourne, Victoria, Australia; University of Minnesota Twin Cities, UNITED STATES

## Abstract

Although several computational modelling studies have investigated the conformational behaviour of inherently disordered protein (IDP) amylin, discrepancies in identifying its preferred solution conformations still exist between various forcefields and sampling methods used. Human islet amyloid polypeptide has long been a subject of research, both experimentally and theoretically, as the aggregation of this protein is believed to be the lead cause of type-II diabetes. In this work, we present a systematic forcefield assessment using one of the most advanced non-biased sampling techniques, Replica Exchange with Solute Tempering (REST2), by comparing the secondary structure preferences of monomeric amylin in solution. This study also aims to determine the ability of common forcefields to sample a transition of the protein from a helical membrane bound conformation into the disordered solution state of amylin. Our results demonstrated that the CHARMM22* forcefield showed the best ability to sample multiple conformational states inherent for amylin. It is revealed that REST2 yielded results qualitatively consistent with experiments and in quantitative agreement with other sampling methods, however far more computationally efficiently and without any bias. Therefore, combining an unbiased sampling technique such as REST2 with a vigorous forcefield testing could be suggested as an important step in developing an efficient and robust strategy for simulating IDPs.

## Introduction

With advances in computational power, the use of detailed atomistic simulations of biological systems has led to a level of understanding that is not achievable by experimental techniques alone.[1–5] Broadly speaking, there are two important requirements that must be satisfied in order to accurately describe the behaviour of biomolecules using computational modelling.[6] First, it is important to have an appropriate forcefield that accurately represents the interactions between atoms within a biomolecular system. Second, sufficient sampling of the potential energy landscape is required to obtain accurate description of the kinetic and thermodynamic properties of the system. The latter of the two is currently one of the main drawbacks of classical molecular dynamics (MD) applications to biomolecular systems. The sampling deficiency is omnipresent when simulating intrinsically disordered proteins (IDP), since it is essential for these systems to be able to efficiently cross energy barriers between local energy minima.[7] Currently, comprehensive conformational sampling using brute-force MD alone is only achievable on specially designed hardware like Anton designed and purpose build by D.E. Shaw Research.[8]

**Fig 1:**
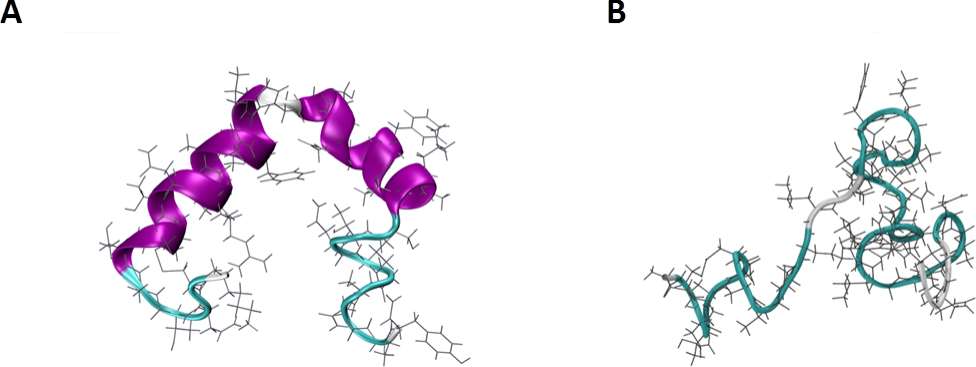
Cartoon/licorice representation of the starting structures of human amylin used in this work. (A) NMR micelle bound structure PDB code 2L86.^[^^15^^]^. (B) Unfolded random coil conformation (taken from our preliminary assessment of amylin).

One important example of an intrinsically disordered protein is the human islet polypeptide (hIAPP), or amylin, which is co-expressed and co-secreted alongside insulin in the pancreatic β-cells. However, when functional amylin proteins misfold they convert into insoluble amyloidogenic fibril-like structures.[9] This disorder is classified as a form of metabolic conformational disease, which had been strongly implicated in the development of type-II diabetes mellitus. [10, 11] Detailed understanding of the conversion process of functional proteins into toxic amyloid fibrils could aid the development of targeted medicinal applications and novel therapeutics. Nonetheless, the structure of monomeric amylin has been extensively studied using experimental techniques; including circular dichroism and solid-state NMR which have shown amylin can adopt a variety of random coil, β-hairpin and α-helical structures.[12–14] Nanga et al. determined a helix-turn-helix conformation of amylin when bound to a micelle (Fig 1A).[15] However, Goldsbury et al. showed the aggregation of amylin to amyloidogenic fibrils involved a conformational change from random coil (Fig 1B) to β-sheet/α-helical structures. Therefore, an appropriate computational model, including both the conformational sampling method and forcefield, must be capable to reflect these conformational ranges.[12]

As with any protein, it is necessary to gain a comprehensive understanding of its preferred conformational states in solution and classical MD simulations are typically the tool to be used, however a comprehensive conformational sampling is not achievable using brute-force MD, without sacrificing a great amount of time and computational resources.[16] As a result, a range of enhanced sampling methods have been developed in the past decade to overcome the limitations of the brute-force approach including replica exchange molecular dynamics (REMD [17] or T-REMD [18]), bias-exchange metadynamics (BEMD)[19] and, more recently, replica exchange with solute tempering (REST2).[20] In the REMD method, several non-interacting replicas of the same system are simulated at different temperatures. At selected exchange times, a Monte Carlo exchange is performed between the replicas, with the exchange of configuration accepted or rejected based on a Metropolis acceptance criterion. [17, 21] This enables the higher temperature replicas to cross energy barriers, while the low temperature replicas sample larger conformational space. The main drawback of REMD is that a large number of replicas are required to obtain effective sampling, which can be computationally expensive. Another advanced sampling method, bias-exchange metadynamics (BEMD), relies on history-dependent biases to overcome free-energy barriers. In this approach, the choice of collective variables is crucial for maintaining the accuracy of system modelled; if slow changing degrees of freedom are not included, inaccurate systematic biases in free-energy can occur. [17, 22] A relatively new advanced sampling technique based on replica exchange with solute tempering is designed to increase the efficiency of protein sampling in aqueous solution by transforming the Hamiltonian of each replica (instead of using different temperatures), and is also known as Hamiltonian-REM. Liu et al. developed this method to only modulate the solute in the simulation, thus increases in energy are only applied to part of the system. This makes replica exchange with solute tempering (revised and renamed as REST2 in [23] capable of advanced sampling using a small number of replicas, and therefore is more computationally efficient relative to other replica exchange methods. [20, 24]

The conformational behaviour of monomeric amylin has been previously investigated using different advanced sampling techniques. Most recently, Hoffman et al. used BEMD to study the forcefield effects on the structure and dynamics of rat and human amylin to determine which forcefield was most suitable to model the species-related conformational differences.[5] Their work highlighted the structural preferences (biases) of specific forcefields, where GROMOS forcefields, in general, exhibited a conformational bias towards hairpin structures, CHARMM27 towards α-helices, OPLS-AA/L towards random coil, while Amberff03w and CHARMM22* provided a balance between secondary structures consistent with available experimental literature.[5] Furthermore, Zerze et al. provided a comparative analysis of the hIAPP conformational sampling using both T-REMD and BEMD to elucidate the differences between the two methodologies.[4] Results from this study determined that both techniques yielded consistent results based on the free-energy and secondary structure analysis. However, the analyses highlighted that the selection of collective variables in BEMD has to be taken with caution, as lower helical propensity resulted from BEMD compared to T-REMD sampling due to the exclusion of residues 2–7 from the α-RMSD bias. Nonetheless, the studies showed the ability of BEMD and T-REMD to efficiently sample the conformational landscape of intrinsically disordered proteins and provided the necessary benchmarking data for an independent assessment of REST2, which can alleviate the computational expenses and biases associated with T-REMD and BEMD [4]. In this work we used the membrane bound conformation of amylin as starting structure to determine the ability of common forcefields to take the protein out of the helical conformation into the disordered solution state as seen in experiment [12]. Additional simulations starting from the unfolded random coil conformation of amylin were also performed to enhance conformational sampling and elucidate any forcefield related biases. We systematically compare the conformational preferences exhibited by these forcefields using brute-force MD and non-biased sampling techniques such as REST2, with other similar simulations studies [4, 5].

## Methodologies

### Classical MD simulations

All simulations were carried out using the GROMACS 5.0.5 software package.[25] Classical “brute-force” MD and REST2 simulations were performed on two different starting structures, the native NMR structure of hIAPP (PDB code 2L86) [15] and unfolded random coil using five different forcefields, listed in Table 1. All forcefields used in this study are available in the GROMACS simulations package. The peptide was modelled in zwitterionic form with NH^3+^ and COO^-^ as termini. The protein was enclosed in a periodic box of 65 Å x 65 Å x 65 Å size, and solvated with 8900 TIP3P, TIP3SP or SPC waters in accord with the forcefield applied to achieve the water density of 1 g/cm^3^.[3] To investigate the role of the water model in the protein conformational dynamics, the CHARMM22* and CHARMM27 simulations were also repeated with the modified TIP3P water model (TIP3SP). Two Cl^-^ counter ions were added to the system to neutralise the overall +2 charge. Van der Waals and electrostatic interactions were truncated at 1 nm with long-range electrostatics calculated by Particle Mesh Ewald summation method.[26] Energy minimisation was initially carried out to remove any steric clashes using steepest descent algorithm until convergence was achieved at 250 kJ/mol. This was followed by three equilibration stages: (1) 100 ps NVT (constant volume/temperature) simulation to relax the system, (2) 2 ns simulation having the solute position restrained to relax the solvent around the protein and (3) 10 ns of un-restrained NPT (constant pressure/temperature) simulation. Constant pressure of 1 bar was achieved by coupling the system using the Berendsen barostat, with coupling constant of 2.0 ps and reference pressure of 1.0 bar,[27] and constant temperature of 300 K and coupling constant of 0.1 ps was achieved using the v-rescale [28] method for the course of the simulation. The LINCS algorithm [29] was applied to constrain the bond lengths to their equilibrium values which enabled a simulation time-step of 2 fs to be used. Following this preparation, the systems were subjected to data collection by brute force MD (for comparison purposes) and REST2 simulations, described below and summarised in Table 1. Two concurrent simulations were run with different starting velocities for the brute force MD simulations.

**Table 1:** Total simulation times collected for each forcefield employed and respective water models. Convergence of the REST2 simulations was determined using cluster analysis, whilst brute-force MD simulation was considered to reach equilibrium when the system energies and backbone RMSD had plateaued. CHARMM22/CMAP is designated as CHARMM27.

Forcefield (FF)	Water Model	Simulation Length (ns)			
		Folded		Unfolded	
		MD	REST2	MD	REST2
AMBER99SB*-ILDN [31]	TIP3P	600	100	500	30
**GROMOS96 54a7 [32]**	SPC	600	200	500	30
**CHARMM36 [33]**	TIP3SP	500	100	500	30
CHARMM22* [30]	TIP3P/TIP3SP	500	80	500	30
**CHARMM27 [34]**	TIP3P/TIP3SP	500	80	500	30

### REST2 simulations

For the REST2 simulations, the equilibrated conformation of amylin was used as a starting structure with 16 replicas for each forcefield, except for CHARMM36, where 24 replicas were used (the reason for this is discussed later). Van der Waals and electrostatic interactions were kept consistent with the classical MD settings described above.

The free-energy perturbation and replica exchange code within GROMACS was used to implement the REST2 methodology as outlined by Terakawa et al.[20] and re-examined by the original REST developers, Berne et al. [23], also known as REST2. The effective temperatures for each system were set within the interval of 300 to 600 K. The average probability, λ, for an attempted exchange was approximately 0.5, using a series of 1 ns REST runs, and an exchange was attempted every 1000 MD steps. The total simulation times for each forcefield using REST2 are listed in Table 1. Each REST2 simulation was determined as converged when the clustering of the neutral (unbiased) replica trajectory reached a plateau (i.e. new significant clusters were no longer forming). This approach has been applied in other similar studies using REST2.[35] All analyses of the REST2 simulations were conducted on the neutral replica.

#### Data analysis

Analyses of the simulation data was performed on the final 50 ns of the converged trajectory for the MD simulations, and 40 ns for the neutral REST2 replica. The convergence of the REST2 simulations was confirmed by monitoring the development of new highly populated clusters over the last 40 ns of simulations. [35] The convergence of the MD simulations was determined when the system energies and RMSD of the protein backbone had reached a plateau. Here, the plateauing RMSD was used as a criterion for stopping the MD simulation runs considered conformationally trapped and not useful to continue. Due to the nature of classical molecular dynamics simulations, it is not feasible to match MD simulated time to that of REST2, thus classical MD simulation results are only indicative of the limitation of this sampling method. Clustering analysis using the LINKAGE method [36] was first applied to determine the most populated structures during the equilibrated period of the simulation. To obtain the most populated clusters, where ~50% of the sampled structures are found in the top 3 clusters, a different RMSD cut-off was applied for each sampling method. Specifically, a cut-off of 2 Å was used for the REST2 simulations to capture a high variation of conformations sampled, compared to the 1.5 Å cut-off used for the more conformationally restricted brute-force MD generated trajectories. The protein secondary structure content sampled in each simulation was calculated using the STRIDE algorithm in VMD.[37] The average number of residues that adopted a particular secondary structure element (coils, turns, helices and β-strands) was calculated. The three types of helical structures, α-helix, π-helix and 3_10_-helix were summed together as the helical group. Similarly, strands were defined by the combination of β-bridges and β-sheets. In addition, the software code PLUMED 2.0 [38] was used to construct conformational free-energy landscape sampled by each forcefield starting from the folded conformation using the REST2 and MD (shown in Supporting Information S3 Fig).

## Results and discussion

### Conformational dynamics

**Fig 2:**
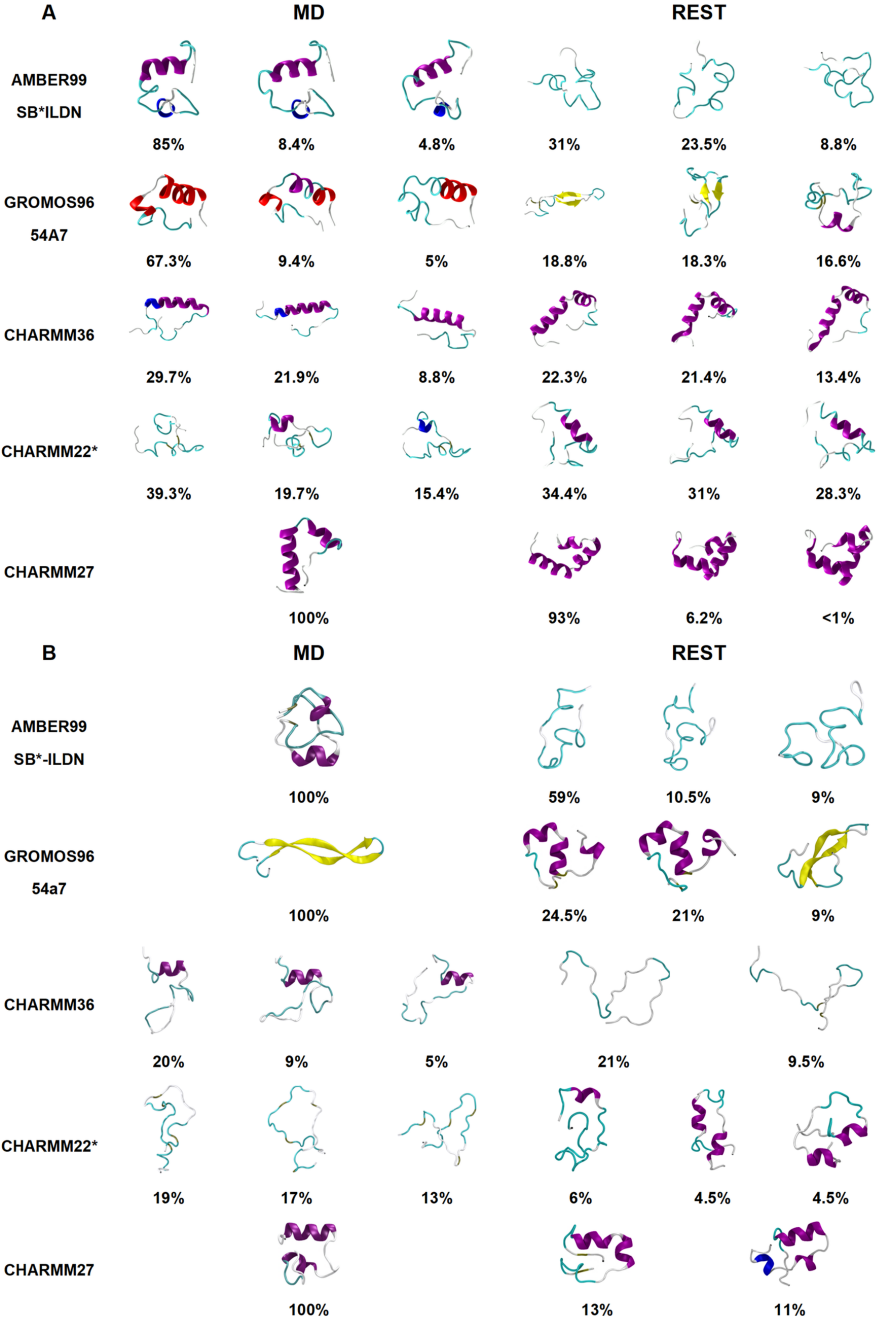
Clustering analysis results from MD and REST2 simulations starting from (A) folded NMR structure and (B) disordered (unfolded) state of amylin. Top clusters for each simulation are selected to represent the most common conformations of the equilibrated trajectory. The protein secondary structure is represented as cartoon with the α-helix coloured in purple, 3-10-helix in blue, turn in cyan and coil in white and extended β-sheet in yellow.

**Fig 3:**
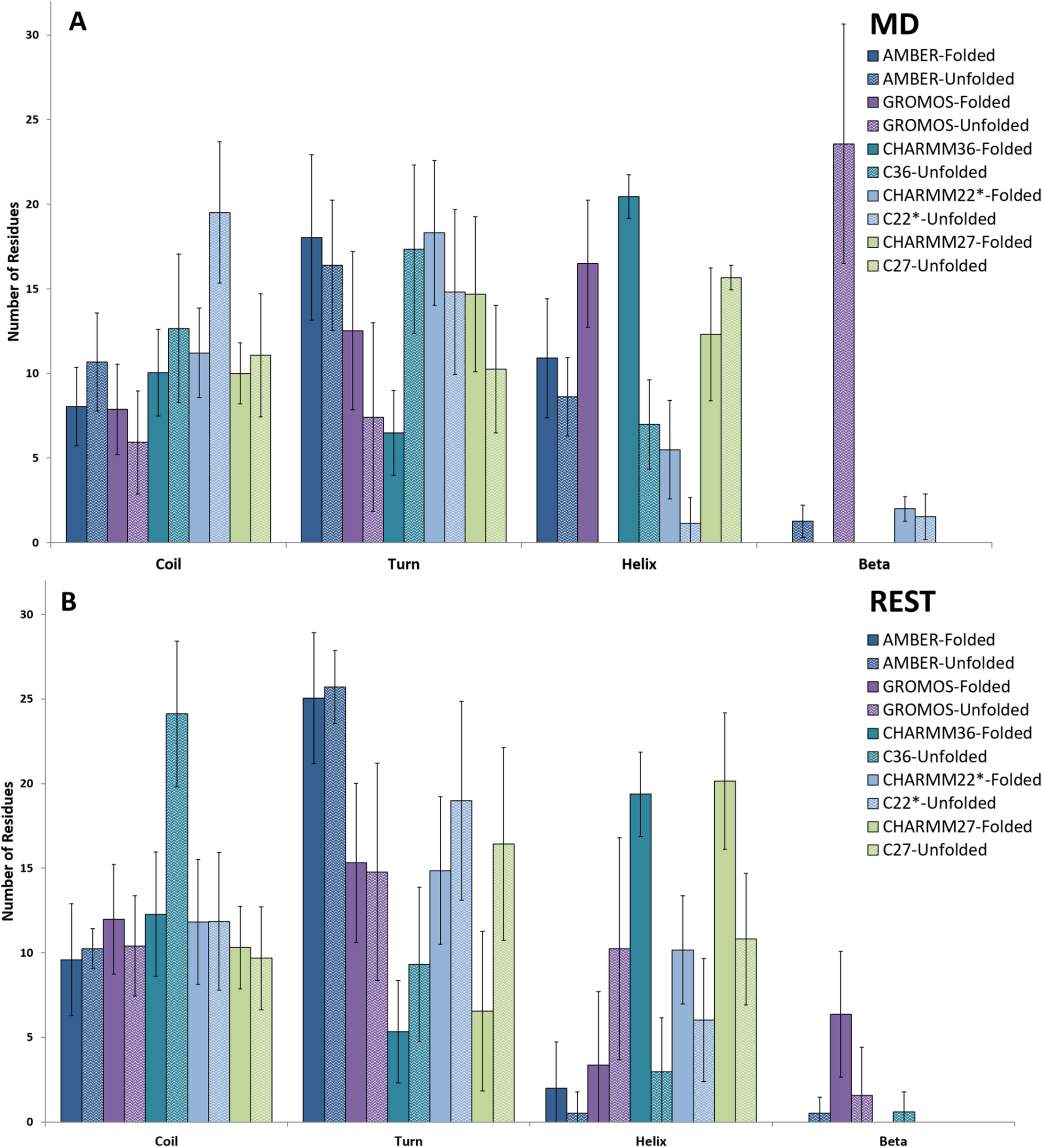
Average number of residues showing each secondary structure element determined on the equilibrated period of the (A) MD and (B) REST2 simulations for each forcefield and starting structure. The pattern filled bars are representing the unfolded runs while the solid filled bars with the same colour are the folded runs.

Clustering and secondary structure analyses were performed over the equilibrated period of both brute-force MD and REST2 simulations starting from the folded (mostly helical) and unfolded (random coil) conformations. The results are presented in Figs 2 and 3, respectively. The α-helical versus β-strand content sampled by each forcefield was also determined using PLUMED, (Supporting Information S3 Fig). The MD simulations starting from the folded conformation using the AMBER99SB*-ILDN forcefield exhibited stable conformations, most populated cluster having 85% of equilibrium structures, with some helical content between residues 5–16 and coils along the amyloidogenic C-terminus. Similarly, the simulations starting from the unfolded conformation produced a single cluster (100% population) albeit with a slightly less helical content (~8 residues) with short helical segments along both amyloidogenic sections of the protein. In contrast, the REST2 simulations with the AMBER99SB*-ILDN forcefield, exhibited mostly disordered states dominated by turn and coil conformations, irrespective of the starting structure. Different conformational behaviour was observed in the simulations using the GROMOS96 54a7 forcefield. The brute-force MD simulation starting from the folded amylin conformation resulted in a stable helical structure (~67% population), with ~16 residues on average forming helices and the REST2 forming coils and turns. While the simulation starting from an unfolded amylin showed a 100% dominant cluster composed of β-hairpin-like conformations, as seen in the secondary structure results in Fig 3, where ~24 residues were in the β-strand conformation. Interestingly, this observation was reversed in the REST2 simulations with GROMOS96 54a7 forcefield, albeit the overall reduced β-strand/helical structure content. The two most populated clusters from the simulations starting from the folded structure consisted of a ~6 residue in β–strand conformation, while the remaining clusters with lower populations contained some helical (<5 residues) and random coil conformations. The REST2 simulations starting from the unfolded structure exhibited ~46% of structures with shorter helices spread across ~10 residues along the amyloidogenic segments of the protein, whereas there were only low populated clusters exhibiting some β-structuring less than 5 residues on average. The CHARMM forcefield MD simulations starting from the folded conformation presented mostly helical conformations, with the exception of CHARMM22*. The CHARMM36 simulations displayed clusters with extended helices and flexible termini where on average ~20 residues were in a helical conformation during the equilibrated period while CHARMM27 produced a single cluster resembling the NMR micelle bound configuration with ~13 residues in helical conformation. The brute-force MD trajectories using CHARMM22* forcefield exhibited short 3–5 residue helical segments, present in 60% of the clusters formed, whilst the simulations starting from an unfolded structure remained fully disordered. The CHARMM36 simulations starting from the unfolded structure exhibited a small helical formation ~6 residues on average, while the CHARMM27 forcefield was able to fold back the protein into a mostly helical structure with ~16 residues adopting helix-like conformations. This result illustrates that brute-force MD is unable to overcome the helical propensities of the CHARMM27 forcefield, as seen in other similar studies [4, 5]. In the REST2 simulations starting from the folded structure using the CHARMM22* forcefield, the protein formed semi-helical conformations similar to that of brute-force MD results, with a slightly longer helical segment along residues 6–16 and random coil formation along residues 20–29; whilst the unfolded REST2 simulations yielded largely random coil conformations with short 3–5 residue helical segments. Similarly, the brute-force MD, the REST2 simulations starting from folded amylin with CHARMM27 and CHARMM36 forcefields had a majority of the structures forming helix-turn-helix conformations. When CHARMM36 was used to simulate 24 replicas rather than the standard 16 (due to poor exchange rates at higher effective temperature replicas), the highly helical secondary structure was still present which may be indicative of the limitations of the conformational sampling. Conversely, REST2 simulations starting from the disordered state of amylin using the same two CHARMM forcefields resulted in less helical structure formation, with CHARMM36 forming entirely random coils, and CHARMM27 forming ~11 residue helices as opposed to the folded (helical) simulations. These variances between clusters formed by changing the starting structure illustrate two things. Firstly, in brute-force MD, even by using a fully disordered starting structure, certain forcefields (GROMOS96 54a7 and CHARMM27) showed conformational bias towards a specific secondary structure. Secondly, When REST2 is applied to the unfolded starting structure, previously observed forcefield biases were alleviated across all three CHARMM forcefields tested, which highlights the importance of proper sampling with any forcefield.

Overall, the additional simulations starting from the unfolded structure provided valuable observations into the conformational biases of some forcefields and ways to alleviate them. The brute-force MD results suggested that even though starting from an unfolded conformation, GROMOS96 54a7 favoured the **β**-hairpin conformation, while CHARMM27 formed the helix-turn-helix structure resembling the micelle bound NMR conformation. AMBER99SB*-ILDN produced a structure with helical content at either terminus, while CHARMM36 produced a short 3–5 residue helical segment and random coils for the REST2 of the residues. Alternatively, CHARMM22* maintained an overall disordered conformation. REST2 was successful at preventing the protein from being trapped in low energy states and was able to sample a wider conformational space. However, when REST2 was applied to an already folded structure in conjunction with GROMOS96 54a7 and CHARMM27 it was unable to take the conformation out of the low-energy wells, as seen from our simulations. Clearly, the starting structure influenced the resulting conformations despite the extensive conformational sampling. However, the purpose of this study was to investigate the ability of common forcefields to take the protein out of the helical conformation into the disordered solution state as seen in experimentally. We have shown that REST2 is able to mitigate some of the conformational biases exhibited by the forcefields, provided that the starting structure is not already folded in line with the forcefield’s own preferences.

#### Comparison to other forcefield and enhanced-sampling studies

In comparison to the REMD and BEMD results from Zerze et al. using the Amberff03w forcefield, our simulations using REST2 with AMBER99SB*-ILDN yielded slight variations in secondary structures obtained. Whilst these two AMBER forcefields are very different from one another, our results demonstrate that drastic differences persist within different versions of the same family forcefield. AMBER99SB*-ILDN, originating from the ff99SB and ff03 forcefields, have a combination of improved side-chain torsion potentials for four amino acids (-ILDN), and a modification to the backbone dihedral potentials (ff99SB*) to provide better energetic balance between helix and coil conformations. [31, 39] Amberff03w, on the other hand, was derived from Amberff03 with the TIP4P water model, with a small backbone modification to match the population of helical states to experiment. [40, 41] These differences in modifications to the forcefield partly contribute to the variance of sampled conformations between these studies. Amberff03w in REMD and BEMD simulations produced more helical and coil content (13–15 residues as opposed to 10 using REST2, see supporting information S1 Fig; however far less turns (13 residues as opposed to 25 residues seen in our study). Amberff03w also featured β-structure formation (~2 residues) consistent between REMD and BEMD. This was not the case for our REST2 and MD simulations with AMBER99SB*-ILDN where REST2 resulted in an overall random coil conformation, whilst some helical content (~7 residues) remained within the timeframe used in the brute-force MD simulation. Since the Amberff03w forcefield was derived from Amberff03 with the TIP4P water model, this forcefield was found to produce more cooperative helix-coil transitions relative to the forcefield it was based on.[41] Nonetheless, the observed differences in sampled structures further illustrate the importance of using advanced sampling methods when modelling IDPs. On the other hand, the AMBER99SB forcefield used by Qiao et al. [16] obtained similar secondary structures to our AMBER99SB*-ILDN results. The secondary structures formed in their REMD study showed that approximately 16 residues were in a random coil conformation, 15 residues in bend or turn conformation, 3 in helical conformations and 3 in β-conformation. This is fairly consistent with the results we obtained in our AMBER99SB*-ILDN forcefield study. In a different forcefield study, Dupuis et al. found that the AMBER ff96 forcefield and REMD of human amylin with +3 and +4 protonated states formed significantly more β-sheets than other studies using only the +2 protonated state. [10] Their results showed approximately 10–13 residues in β-sheet conformations for +3 and +4 hIAPP respectively. Only a small portion of residues were in helical conformations, (~7 residues), with the rest making up turns and coiled conformations. Therefore, in addition to forcefield and water models, the protonation state of the protein also plays a significant role in the ambient conformation of a protein. For the purposes of this study, we had chosen the +2 protonation state.

In two separate studies, Hoffman et al. and Zerze et al. utilised the Amberff03w forcefield with the BEMD method to investigate the conformational behaviour of amylin. Their secondary structure results showed minor differences in helical versus strand content despite using the same technique and forcefield (Supporting Information S1 Fig). This variation demonstrates the importance of selection and definition of appropriate collective variables in BEMD simulations, where biases towards one or another secondary structure can play an important role in the conformations formed and sampled. It is also worth noting that Hoffman et al. used different water models for their choice of forcefields. Specifically, the TIP4P[42] model was used for the Amberff03w and CHARMM22* simulations, while TIP3P[43] and SPC[44] water models were used for AMBER99SB*-ILDN and GROMOS96 53a6 respectively. The TIP4P water model was proposed in 2005 as a rigid four site model consisting of three fixed point charges and a single LJ centre with a negatively charged dummy atom along the HOH bisector. While this improves the electrostatic description of the molecule, it renders the model less computationally efficient relative to TIP3P.[42, 43] Recently, an updated version of TIP4P was developed by Piana et al.[45] and its effectiveness on IDPs were tested on Histatin 5 using the AMBER99SB-ILDN forcefield. [46] Here, to investigate the role of the water model on the conformational preferences of folded amylin, brute-force MD and REST2 simulations were repeated using the CHARMM22* and CHARMM27 forcefields and the modified TIP3P (TIP3SP) water model (see Supporting Information S2 Fig). The secondary structure analysis showed the protein had slightly more helical content in the CHARMM27/TIP3SP MD and REST2 simulations, as well as the CHARMM22* REST2 simulations, compared to the simulations using the standard TIP3P water model. However, the TIP3SP CHARMM22* MD simulations, had no observed helical formations, as opposed to the ~5 residue helix seen in standard TIP3P. Nevertheless, the results demonstrated no significant differences between the structures obtained, apart from the small increase in helical content observed in the REST2 simulations. In a separate study, Boonstra et al. [47] suggested the use of unmodified TIP3P water model for modelling disordered proteins, due to a) the minor structural differences observed between the two water models for an intrinsically disordered protein and b) the reduction in computational cost. Following this, the unmodified TIP3P was chosen for testing water models in this study. Furthermore, as Henriques et al. [46] highlighted an accurate protein-water interaction was important for modelling unfolded IDPs and possibly the discrepancies between previous studies [4, 5] and our results would be attributed to the differences between water models used. In addition, a more recent CHARMM36m forcefield was proposed by Huang et al. [48] which improve upon the accuracy in generating backbone conformational ensembles for intrinsically disordered peptides and proteins. It is important to point that this study postulated that there is no single universal parameter applicable to all IDPs, and that IDP compatible water models may provide more accurate results.

### Comparison between REST2 and BEMD

Numerous studies in the past had investigated the conformation of monomeric human amylin using various computational and experimental techniques.[1, 4, 5, 9–11, 49, 50] REMD and BEMD techniques previously employed by Zerze et al. and Hoffman et al. are good examples of applying advanced sampling methods to overcome the known limitations of spontaneous MD simulations, especially when dealing with IDPs. REST2 simulations from this study provide additional quantitative comparison of the advanced sampling approaches for modelling IDPs.

Metadynamics based approaches involve Gaussian potentials added to the forcefield to adaptively bias the MD simulation along predetermined reaction coordinates. These reaction coordinates are classified as the Collective Variables (CVs), typically including α-RMSD, β-RMSD, number of hydrogen bonds, or the number of atomic contacts in a biomolecule, to name a few. Bias-exchange metadynamics accelerates the sampling of metadynamics by using a series of replicas under the control of different biasing potentials and adapting them as the simulation proceeds.[4] On the other hand, REST2 achieves efficient sampling by applying a scaling factor to the potential energy of each replica, as well as reducing the amount of solvent sampling by only tempering the solute of the system.

It has been argued that the choice of CVs is the most important factor in determining the convergence and efficiency of the free-energy calculations in BEMD. [4, 5] Thus, if the chosen CVs are unable to capture the slowest degree of freedom to allow for conformational transitions, or are unable to distinguish between different metastable states, not all regions of the conformational space will be explored. This phenomenon of BEMD simulations is known as hysteresis.[4] Therefore, one would need some preliminary knowledge of the topological, chemical and physical properties of the system. This can, in principle, be overcome by using a very large group of CVs, effectively reducing the importance of selecting the “appropriate” CVs, although this will significantly increase the computational cost.[4] In contrast, in REST2 simulations, the most important factors are the number of replicas used and the highest effective temperature of the solute. Interestingly, a majority of the secondary structures sampled by our REST2 simulations agreed with the results of Zerze et al. and Hoffman et al., with the exception to CHARMM22* and GROMOS96 54a7, although Hoffman et al. used a slightly older variant of the GROMOS forcefield. The 54a7 version of the GROMOS forcefield used here incorporates a new helical propensity adjustment via the torsion term. This may be attributed to the higher helical content in monomeric amylin observed in our simulations starting from the folded amylin structure, compared to an overall β-hairpin structure sampled with 53a6 forcefield.[5] Furthermore, it is important to point out that the studies reviewed here have not considered different starting structures for their simulations, thus any dependence or bias from the initial conformation was not explored. Our REST2 simulations results clearly demonstrated that starting from unfolded conformation REST2 was able to alleviate some of the forcefield biases associated with CHARMM27 and GROMOS96 54a7 forcefields.

To complement the analyses of the sampling abilities of the techniques and the forcefield biases, the final point of comparison is the computational cost of each methodology. T-REMD and BEMD simulations by Zerze et al. were run for 8 μs and 2.4 μs each or 200 ns and 600 ns per replica respectively (total of 40 replicas). On the other hand, Hoffman et al. ran their BEMD simulations for 600 ns for all forcefields except AMBER99SB*-ILDN which was ran for 1 μs. In contrast, the folded REST2 simulations in this work were ran for 100 ns per replica, with the exception to GROMOS96 54a7 that ran for 200 ns per replica; effectively resulting in a simulation time of 1.6 μs and 3.2 μs respectively. Conversely, the REST2 simulations staring from the unfolded state ran for 30 ns per replica, resulted in an effective sampling time of 480 ns, closely matching the simulated time of the respective MD runs. The results from this study indicated that for some forcefield REST2 simulations converged to different structures when performed from the unfolded state. Therefore, REST2 remains more efficient relative to other sampling techniques due to the demonstrated improvements in sampling conformational space in conjunction with potentially biasing forcefields.

## Conclusion

In order to appropriately sample the free-energy landscape of an intrinsically disordered protein, one must perform a systematic analysis utilising the capabilities of advanced sampling techniques and a forcefield capable of reflecting the conformational preferences of such a protein. [3] REST2 simulations were benchmarked against spontaneous MD, T-REMD and BEMD approaches to highlight the advantages of REST2 relative to the other sampling methods. As expected, REST2 simulations sampled a much broader conformational space than brute force MD simulations while yielding agreeable results with MD, T-REMD and BEMD simulations across most forcefields. However, the conformational biases exhibited by some commonly used forcefields appear to persist in simulations by brute-force MD and even by advanced sampling technique such as REST2. By utilising REST2 in a systematic approach using different starting structures we demonstrated that it is possible to improve the sampling of the conformational landscape of an intrinsically disordered protein such as amylin. Starting from the folded and unfolded conformations, the CHARMM27 forcefield favoured helical conformations, whereas CHARMM36 only sampled helical conformations when simulations were initiated from the folded structure. The GROMOS96 54a7 forcefield caused amylin to form some β-hairpin structures as observed in previous studies [5] and the AMBER99SB*-ILDN forcefield, resulted in a fully disordered conformation. Finally, CHARMM22* produced results in best agreement with experimental data, [12–14] providing a balance between disordered and helical conformations for amylin in solution, as experimental results indicated. [12] Overall, CHARMM22* is shown to be less dependent on the starting structure or sampling method in its ability to reproduce experimentally observed solution structures of amylin. The observed agreement with previous computational studies and experiments highlighted the ability of REST2 to explore the wider conformational space of amylin efficiently and effectively, compared to brute-force MD. However, it is important to note that qualitative experimental consistency of the simulation results from advanced sampling methods such as REST2 can be affected by the chosen forcefield, and no one forcefield should be considered universal for all IDPs.

## Supporting information

S1 Fig Average number of residues showing each secondary structure element. This is determined on the equilibrated period of the folded REST2 simulations for each forcefield. §Data taken from Zerze et al. (4) §§Data taken from Hoffman et al (5).
(DOCX)

S2 Fig **Secondary structure calculations for (A) MD and (B) REST2 simulation runs conducted with the modified TIP3SP water model.** The average number of residues showing each secondary structure element determined over the equilibrated period.
(DOCX)

S3 Fig Free energy calculations for human amylin. This was illustrated as a function of α and β RMSD for each of the forcefields investigated in this study starting from the folded conformation. REST2 free-energy plots are on the left-hand side, and MD free-energy plots are on the right hand side.
(DOCX)

S1 Table Clustering structures from TIP3SP simulations. The most favourable conformations and their population determined from clustering analysis over the last 50 ns (MD) and last 5 ns (REST2) of the CHARMM22* and CHARMM27 simulations with the TIP3SP water model. The protein secondary structure is represented as cartoon with the α-helix coloured in purple, 3-10-helix in blue, turn in cyan and coil in white and extended β-sheet in yellow.
(DOCX)
