# Supplementary material for: Effects of forcefield and sampling method in all-atom simulations of inherently disordered proteins: Application to conformational preferences of human amylin
Source: PLoS One. 2017 Oct 12;12(10):e0186219. doi: 10.1371/journal.pone.0186219 (PMC5638406; doi:10.1371/journal.pone.0186219)
Supplement: S1 Fig — This is determined on the equilibrated period of the folded REST2 simulations for each forcefield. §Data taken from Zerze et al. (4) §§Data taken from Hoffman et al (5). (DOCX) [file pone.0186219.s001.docx]

**S1 Fig**

Effects of Forcefield and Sampling Method in All-atom Simulations of Inherently Disordered Proteins: Application to Conformational Preferences of Human Amylin

Enxi Peng^1^, Nevena Todorova^1^, and Irene Yarovsky^1^*

^1^ School of Engineering, RMIT University, Melbourne, Victoria, Australia.

*Corresponding author

E-mail: [irene.yarovsky@rmit.edu.au](mailto:irene.yarovsky@rmit.edu.au)

**Comparing to BEMD and REMD**

**S1 Figure: Average number of residues showing each secondary structure element.** This was determined on the equilibrated period of the folded REST simulations for each forcefield. §Data taken from Zerze et al. (1) §§Data taken from Hoffman et al (2)

# **References**

1. Zerze GH, Miller CM, Granata D, Mittal J. Free Energy Surface of an Intrinsically Disordered Protein: Comparison between Temperature Replica Exchange Molecular Dynamics and Bias-Exchange Metadynamics. J Chem Theory Comput. 2015;11(6):2776-82.

2. Hoffmann KQ, McGovern M, Chiu C-C, de Pablo JJ. Secondary Structure of Rat and Human Amylin across Force Fields. PloS One. 2015;10(7):e0134091.
