# Supplementary material for: Effects of forcefield and sampling method in all-atom simulations of inherently disordered proteins: Application to conformational preferences of human amylin
Source: PLoS One. 2017 Oct 12;12(10):e0186219. doi: 10.1371/journal.pone.0186219 (PMC5638406; doi:10.1371/journal.pone.0186219)
Supplement: S2 Fig — Secondary structure calculations for (A) MD and (B) REST2 simulation runs conducted with the modified TIP3SP water model. The average number of residues showing each secondary structure element determined over the equilibrated period. (DOCX) [file pone.0186219.s002.docx]

**Supporting Information**

Effects of Forcefield and Sampling Method in All-atom Simulations of Inherently Disordered Proteins: Application to Conformational Preferences of Human Amylin

Enxi Peng^1^, Nevena Todorova^1^, and Irene Yarovsky^1^*

^1^ School of Engineering, RMIT University, Melbourne, Victoria, Australia.

*Corresponding author

E-mail: [irene.yarovsky@rmit.edu.au](mailto:irene.yarovsky@rmit.edu.au)

S2 Figure: Secondary structure calculations for (A) MD and (B) REST simulation runs conducted with the modified TIP3SP water model. The average number of residues showing each secondary structure element determined over the equilibrated period.

The secondary structure analysis showed the protein had slightly more helical content in the CHARMM27/TIP3SP MD and REST simulations, as well as the CHARMM22* REST simulations, compared to the simulations using the standard TIP3P water model. However, the TIP3SP CHARMM22* MD simulations, had no observed helical formations, as opposed to the ~5 residue helix seen in standard TIP3P. Nevertheless, the results demonstrated no significant differences between the structures obtained, apart from the small increase in helical content observed in the REST simulations. In a separate study, Boonstra et al. [1] suggested the use of unmodified TIP3P water model for modelling disordered proteins, due to a) the minor structural differences observed between the two water models for an intrinsically disordered protein and b) the reduction in computational cost. As a result, the unmodified TIP3P was the choice of water model in this study.

# **References**

1. Boonstra S, Onck PR, Giessen E. CHARMM TIP3P Water Model Suppresses Peptide Folding by Solvating the Unfolded State. The journal of physical chemistry B. 2016;120(15):3692-8.
