# Supplementary material for: Effects of forcefield and sampling method in all-atom simulations of inherently disordered proteins: Application to conformational preferences of human amylin
Source: PLoS One. 2017 Oct 12;12(10):e0186219. doi: 10.1371/journal.pone.0186219 (PMC5638406; doi:10.1371/journal.pone.0186219)
Supplement: S3 Fig — This was illustrated as a function of α and β RMSD for each of the forcefields investigated in this study starting from the folded conformation. REST2 free-energy plots are on the left-hand side, and MD free-energy plots are on the right hand side. (DOCX) [file pone.0186219.s003.docx]

**S3 Fig**

Effects of Forcefield and Sampling Method in All-atom Simulations of Inherently Disordered Proteins: Application to Conformational Preferences of Human Amylin

Enxi Peng^1^, Nevena Todorova^1^, and Irene Yarovsky^1^*

^1^ School of Engineering, RMIT University, Melbourne, Victoria, Australia.

*Corresponding author

E-mail: [irene.yarovsky@rmit.edu.au](mailto:irene.yarovsky@rmit.edu.au)

# **Free-Energy Calculations**

Free-energy calculations were employed to illustrate differences in covering the free-energy landscape of amylin in solution between the forcefields and sampling methods. These analyses were carried out only on one (folded) starting structure. The free-energy maps for hIAPP were constructed as a function of α- and β-RMSD, as shown in Figure S3 for the range of forcefields and sampling methods employed in this study. These collective variables are specially constructed by Pietrucci et al. [1] as quantitative measures calculated as defined in the PLUMED software package and used to illustrate the free-energy landscape of secondary structures formed in the simulations. Specifically, for α-RMSD, the number of 6 residue units was counted against an idealized α-helical structure; as a result, this CV is defined as a differential function of the atomic coordinates. This means that a 6 residue sequence, with a secondary structure closely related to the idealised structure, has an α-RMSD value closer to 1. Whilst another sequence that does not resemble an idealised helical structure has an α-RMSD closer to 0. The same procedure was applied to define the β-RMSD. [1] Therefore, both α- and β-RMSDs are proportional to the number of residues in each of the secondary structure conformation. Thus a higher value of α-RMSD suggests that a larger fraction of the protein is in an α-helical state, and this is likewise with β-RMSD.


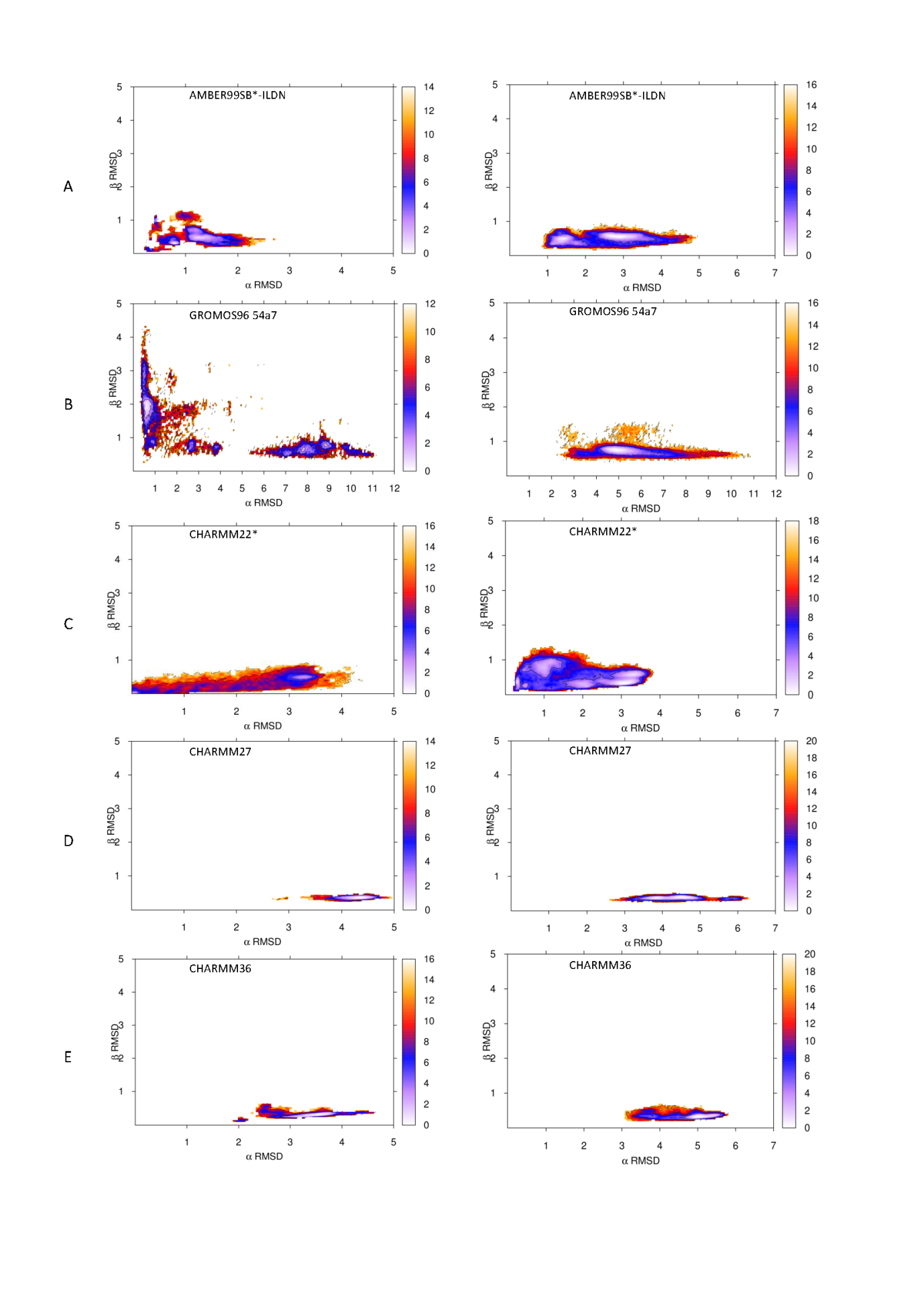


S3 Figure: Secondary structure free energy profiles for human amylin. This was illustrated as a function of α and β RMSD for each of the forcefields investigated in this study starting from the folded conformation. REST free-energy plots are shown on the left and MD free-energy plots are shown on the right.

Figure S3A shows that AMBER99SB*-ILDN simulations resulted in consistently random coil conformations for both simulation methods used (REST on the left and MD on the right), which is similar to the pre-amyloidogenic structures proposed in literature. [2] However, for GROMOS96 54a7 shown in Figure S3B, REST simulations resulted in a distribution of free-energy minima along both α- and β-RMSDs axes. This clearly highlights the different secondary structures sampled (shown in Figure 2), where some clusters formed β-hairpins, and others formed helix-coil conformations. However, in brute force MD, only helix-coil structures were sampled as shown by both the clustering analysis and the free-energy landscape analysis. This attests the ability for REST to sample a wider range of conformation space, which is typically the main limitation of spontaneous MD simulations. Nonetheless, both MD and REST simulations using CHARMM22* were able to sample a wider ensemble of conformations, indicated by the population of multiple free-energy minima during the MD simulation shown in Figure S3C. In line with this, wider distributions of alpha helical and disordered conformations were sampled by the REST simulation, as exemplified clearly by the extended free-energy map along the x-axis. The broad conformational space sampled with CHARMM22* parameters is in line with experimental data that outlines a semi-structured/random coil nature of amylin in solution. [2,3] In contrast, Figure S3D and S3E show highly overestimated helical content when amylin conformations are sampled with the newer CHARMM27 and CHARMM36 forcefields in both classical MD and REST simulations.

# **References**

1. Pietrucci F, Laio A. A collective variable for the efficient exploration of protein beta-sheet structures: Application to SH3 adn GB1. Journal of chemical theory and computation. 2009;5(9):2197-201.

2. Goldsbury C, Goldie K, Pellaud J, Seelig J, Frey P, Muller SA, et al. Amyloid fibril formation from full-length and fragments of amylin. J Struct Biol. 2000;130(2-3):352-62.

3. Yonemoto IT, Kroon, G.J.A., Dyson, H.J., Balch, W.E., Kelly, J.W. Amylin Proprotein Processing Generates Progressively More Amyloidogenic Peptides that Initially Sample the Helical State. Biochemistry. 2008;47(37).
