# Supplementary material for: Effects of forcefield and sampling method in all-atom simulations of inherently disordered proteins: Application to conformational preferences of human amylin
Source: PLoS One. 2017 Oct 12;12(10):e0186219. doi: 10.1371/journal.pone.0186219 (PMC5638406; doi:10.1371/journal.pone.0186219)
Supplement: S1 Table — The most favourable conformations and their population determined from clustering analysis over the last 50 ns (MD) and last 5 ns (REST2) of the CHARMM22* and CHARMM27 simulations with the TIP3SP water model. The protein secondary structure is represented as cartoon with the α-helix coloured in purple, 3-10-helix in blue, turn in cyan and coil in white and extended β-sheet in yellow. (DOCX) [file pone.0186219.s004.docx]

**S1 Table**

Effects of Forcefield and Sampling Method in All-atom Simulations of Inherently Disordered Proteins: Application to Conformational Preferences of Human Amylin

Enxi Peng^1^, Nevena Todorova^1^, and Irene Yarovsky^1^*

^1^ School of Engineering, RMIT University, Melbourne, Victoria, Australia.

*Corresponding author

E-mail: [irene.yarovsky@rmit.edu.au](mailto:irene.yarovsky@rmit.edu.au)

# **Modified TIP3P Water Simulations**

The CHARMM22* and CHARMM27 simulations were also repeated with the modified TIP3P (TIP3SP) water model using both brute-force MD and REST.

S1 Table: Clustering structures from TIP3SP simulations. The most favourable conformations and their population determined from clustering analysis over the last 50 ns (MD) and last 5 ns (REST) of the CHARMM22* and CHARMM27 simulations with the TIP3SP water model. The protein secondary structure is represented as cartoon with the α-helix coloured in purple, 3-10-helix in blue, turn in cyan and coil in white and extended β-sheet in yellow.

|  | **MD** | **REST** | |
| --- | --- | --- | --- |
| **CHARMM22*/TIP3SP** | 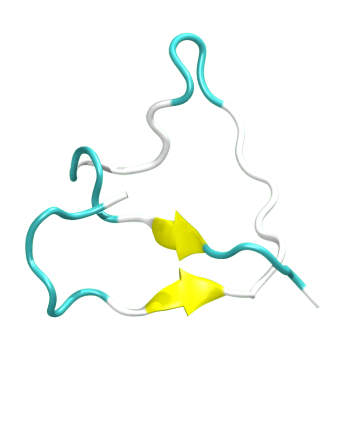 | 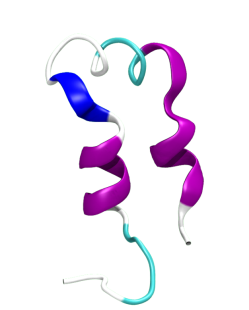 | 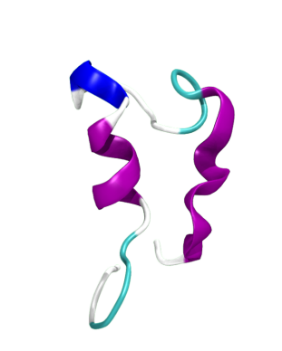 |
|  | 100% | 55% | 24% |
| **CHARMM27/TIP3SP** | 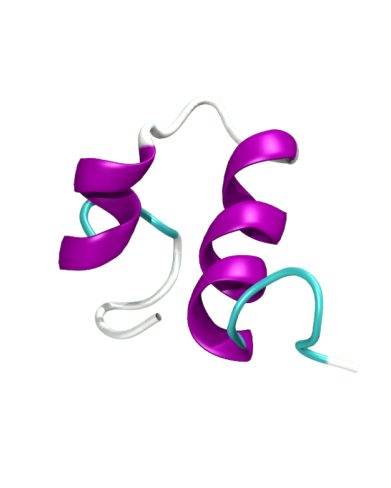 | 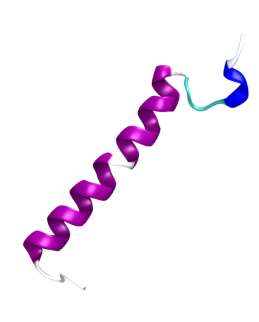 | 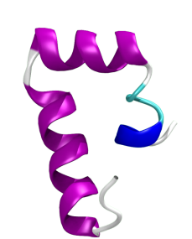 |
|  | 100% | 68% | 18.5% |
